# Niche Partitioning and Facilitative Coexistence of Two Sympatric Pheasants Across a Gradient of Human Disturbance

**DOI:** 10.1002/ece3.73929

**Published:** 2026-07-02

**Authors:** Xiangxiang Lu, Wenbo Yan, Zhigao Zeng, Shaoliang Xue, Qi Wang

**Affiliations:** ^1^ School of Biological Science and Engineering Shaanxi University of Technology Hanzhong China; ^2^ State Key Laboratory of Animal Biodiversity Conservation and Integrated Pest Management, Institute of Zoology Chinese Academy of Sciences Beijing China; ^3^ Hainan Institute of National Park Haikou China; ^4^ Jianfengling Branch Bureau of Hainan Tropical Rainforest National Park Management Office Ledong China

**Keywords:** Hainan peacock‐pheasant, human disturbance, niche partitioning, silver pheasant, species coexistence

## Abstract

How ecologically similar species coexist in human‐impacted tropical islands remains poorly understood. We investigated the spatiotemporal niche partitioning and interspecific interactions between the endangered, endemic Hainan Peacock‐Pheasant (*Polyplectron katsumatae*) and the more common Hainan Silver Pheasant ( *Lophura nycthemera whiteheadi* ) to assess how anthropogenic pressure mediates their coexistence. Using 125 camera traps (28,241 trap‐days) and hierarchical Bayesian occupancy modeling, we quantified their activity patterns, habitat preferences, and spatial associations across a human disturbance gradient in Hainan's tropical montane rainforest. The pheasants exhibited clear temporal segregation: the Peacock‐Pheasant was strictly crepuscular, whereas the Silver Pheasant was diurnal with a single morning peak, and their activity overlap was lower in the wet season than in the dry season. Spatially, they showed contrasting preferences: the Silver Pheasant occupied higher elevations with high NDVI, while the Peacock‐Pheasant preferred mid‐elevations with medium NDVI and proximity to water in the dry season. Despite this niche differentiation, a significant positive spatial association was detected (Species Interaction Factor: 2.48–3.01), indicating co‐occurrence was more frequent than expected by chance. However, human disturbance critically modulated this relationship. The Peacock‐Pheasant was extremely disturbance‐sensitive, its occupancy declining to near zero in areas of high human impact. The Silver Pheasant exhibited greater tolerance, with occupancy peaking at intermediate disturbance levels. Our results demonstrate a coexistence mechanism stabilized by multidimensional niche partitioning but rendered conditional and fragile by anthropogenic pressure. Conservation must prioritize protecting core habitats for the sensitive Peacock‐Pheasant while maintaining landscape connectivity and managing disturbance gradients to preserve this intricate interspecific dynamic.

## Graphical abstract

This study reveals that Hainan Peacock‐Pheasants and Silver Pheasants coexist through distinct temporal and spatial niche partitioning. However, human disturbance disrupts their positive association, as the sensitive Peacock‐Pheasant avoids high‐impact areas while the tolerant Silver Pheasant persists. Consequently, anthropogenic pressure renders their coexistence fragile, requiring conservation strategies focused on habitat protection and disturbance management.

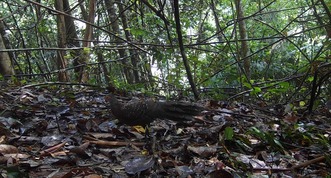

## 1 Introduction

Tropical forests are global biodiversity hotspots, notable not only for high species richness but also for the complex niche‐partitioning mechanisms that enable stable species coexistence (Myers et al. 2000; Wiegand et al. 2025). Understanding these mechanisms is central to explaining community assembly and biodiversity maintenance (MacArthur and Levins 1967). However, logistical challenges such as complex terrain and dense canopy often hinder traditional surveys in these ecosystems, leading to a paucity of ecological data for many rare species and impeding effective conservation (Ahumada et al. 2011; Pimm et al. 2014; Wearn et al. 2017). In this context, terrestrial birds, particularly pheasants (Phasianidae), are an ideal model group for investigating coexistence. They are highly dependent on forest ground microhabitats and are often vulnerable due to long life cycles, limited dispersal, and sensitivity to human disturbance (Liang and Zhang 2011; Zhang et al. 2003). This vulnerability is exacerbated in fragmented tropical island ecosystems (Bennett et al. 2000). Therefore, clarifying how sympatric pheasants coexist is both an important ecological question and a conservation imperative.

The endangered Hainan Peacock‐Pheasant (*Polyplectron katsumatae*) epitomizes these challenges. Endemic to Hainan Island, China, it is restricted to primary tropical montane rainforest within a narrow elevation range (500–1200 m) in central and southern Hainan Island. Its small population is fragmented, and it is listed as Endangered on the IUCN Red List and Critically Endangered on China's Red List, and National First‐Class Protected Wildlife species in China (BirdLife International 2022; Jiang et al. 2016). Despite its precarious status, systematic studies of its ecology remain scarce (Gao and Yu 1990; Li 2005). Its primary sympatric congener is the Hainan Silver Pheasant ( *Lophura nycthemera whiteheadi* ), which is more widespread and classified as National First‐Class Protected Wildlife species in China (BirdLife International 2025; Jiang et al. 2016; Mo et al. 2021). Understanding the interaction between these two species—whether competitive exclusion or niche partitioning prevails—is crucial, especially as habitat loss due to activities like rubber plantation expansion threatens both species (Gao and Yu 1995; Liang et al. 2007).

Coexistence theory, since Hutchinson's (1959) competitive exclusion principle, has evolved to include mechanisms like niche differentiation and spatiotemporal resource partitioning (Chesson 2000; Hubbell 2001). In complex tropical forests, environmental heterogeneity allows species to partition niches through vertical stratification, dietary specialization, and activity rhythms (Orians 1974). While pheasant ecology has been studied in subtropical and temperate regions, empirical work on their coexistence in human‐impacted tropical islands is limited (Hou 2021; Xie et al. 2022; Zhou et al. 2022). Key unanswered questions include: how do these pheasants adjust their spatiotemporal niches to mitigate competition, and how do human disturbances alter their coexistence? This critical information constitutes the missing link connecting ecological theory with conservation practice.

To address these gaps, we conducted a study in the Jianfengling area of Hainan, one of China's best‐preserved tropical montane rainforests (Huang et al. 1986; Zang 2019), focusing on the endangered endemic Hainan Peacock‐Pheasant and Hainan Silver Pheasant. By integrating infrared camera monitoring technology and hierarchical Bayesian occupancy models, we aimed to: (1) quantify niche overlap and differentiation of the two pheasant species along temporal (diel and seasonal) and spatial (elevation, vegetation, water, human disturbance) axes; (2) identify key environmental drivers of their distribution; and (3) assess how human disturbance modulates their interspecific spatial association and coexistence stability. Our findings provide critical insights for the conservation of the Hainan Peacock‐Pheasant and enhance the understanding of coexistence mechanisms in tropical forests.

## 2 Materials and Methods

### 2.1 Study Area

**FIGURE 1:**
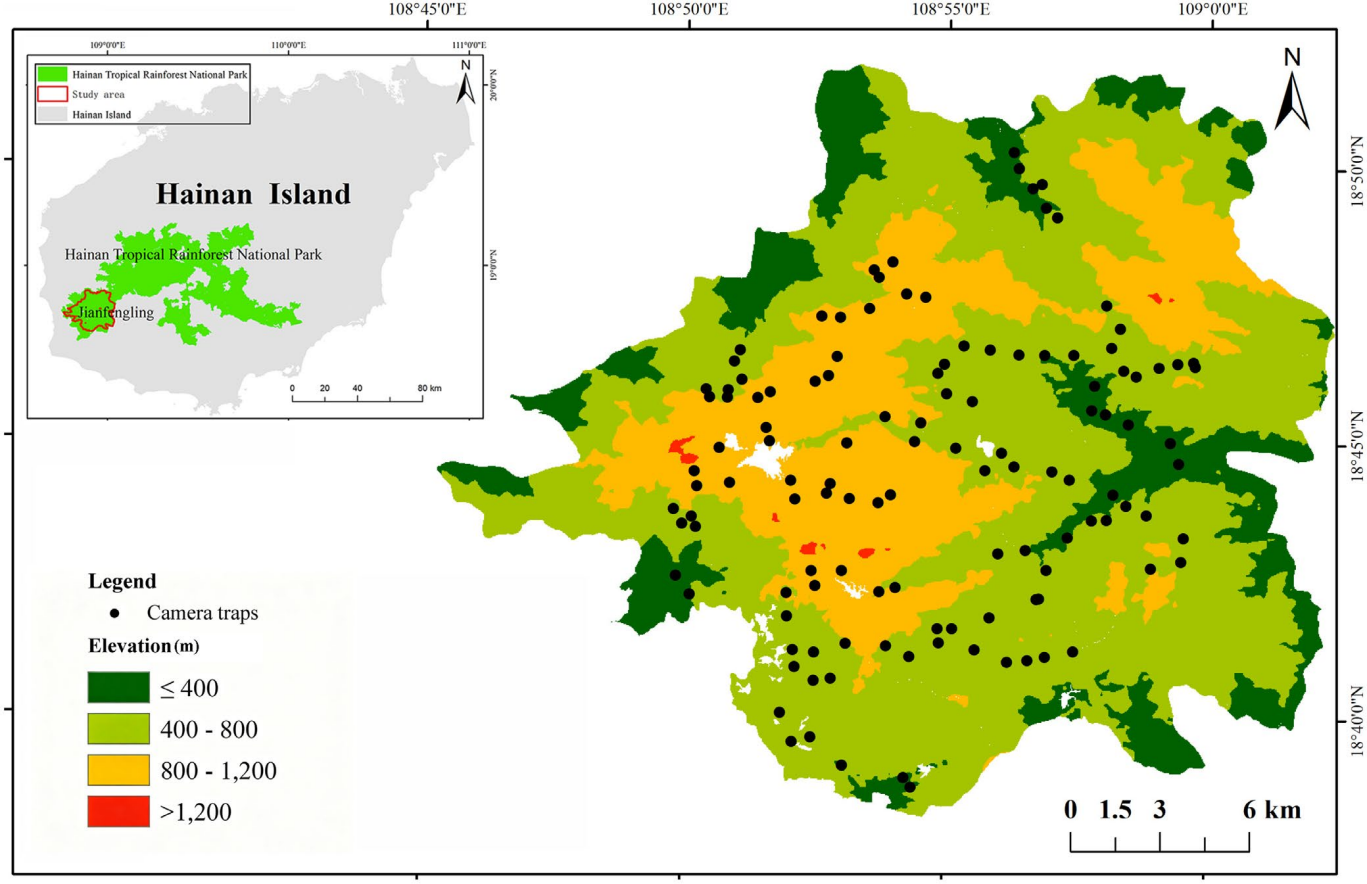
Distribution of infrared camera trap sites in the Jianfengling forest region of Hainan Tropical Rainforest National Park from November 2020 to October 2021.

This study was conducted in the Jianfengling forest region, located in southwestern Hainan Island, China (18°38′ N–18°52′ N, 108°45′ E–109°03′ E), belonging to the Jianfengling section of the Hainan Tropical Rainforest National Park, covering a total area of approximately 640 km^2^ (Figure 1). The region features well‐preserved tropical montane rainforest under a tropical monsoon climate, with mean annual temperatures of 19.7°C–25.0°C and annual precipitation of 2200–3200 mm. A distinct dry season (November–April) and wet season (May–October) occur. The area is a biodiversity hotspot, hosting over 5700 recorded species, including 215 bird species, and is a vital habitat for the focal pheasants (Mo et al. 2021).

### 2.2 Data Collection

The the Jianfengling forest region was divided into 505 grid cells of 1 km × 1 km. We selected 122 grids to deploy camera traps, with at least one infrared camera trap installed in each selected grid. This sampling network adequately covered all elevation gradients and vegetation types within the study region. We deployed a systematic grid of 125 passive infrared camera traps (Ltl Acorn 6511 and E3H series) across the region from November 2020 to October 2021. To reduce spatial autocorrelation and ensure sampling independence, the linear distance between camera sites was greater than 500 m (Figure 1). All cameras were mounted to tree trunks 50–150 cm above the ground. They operated 24 h per day, recording three photos and a 15‐s video per trigger. We serviced cameras quarterly. An independent detection event was defined as all records of the same species at a camera within a 30‐min interval. The survey totaled 28,241 trap‐days, yielding 297 detections of the Hainan Peacock‐Pheasant and 398 of the Silver Pheasant.

### 2.3 Data Analysis

#### 2.3.1 Activity Rhythms

We analyzed diel activity patterns using kernel density estimation in R (“overlap” package “overlapEst” function; R Core Team 2025; Meredith and Ridout 2014). All infrared camera‐recorded times were converted to solar time to correct for the influence of sunrise/sunset variation on activity pattern interpretation (Nouvellet et al. 2012). The overlap coefficient (Δ, range 0–1) quantified the overlap in daily activity density between species, estimated using the Δ₄ index for large samples. Statistical significance was assessed via a bootstrap test (999 iterations).

#### 2.3.2 Occupancy Modeling

We used a Hierarchical Bayesian Multi‐species Occupancy Model (HBOM) to analyze species‐environment relationships (Kéry and Royle 2016). Based on literature and preliminary surveys, we included five covariates: Human Influence Index (HII), Normalized Difference Vegetation Index (NDVI), elevation, aspect, and distance to water source (Li 2005; Li et al. 2009). HII data were obtained from the 2019 Global Human Footprint dataset released by the Wildlife Conservation Society (https://wcshumanfootprint.org). This index integrates multiple sources of information including population density, infrastructure, roads, and nighttime lights (Sanderson et al. 2022). HII values in the Jianfengling forest region ranged from 275 to 4173. Based on our field survey data, HII < 500 was defined as low human disturbance, HII 500–800 as medium disturbance, and HII > 800 as high disturbance. Elevation and aspect data were derived from a 30 m resolution Digital Elevation Model (DEM). NDVI data came from the MODIS MOD13Q1 Version 6 product (spatial resolution 250 m, temporal resolution 16 days). Distance to water source was calculated based on a 1:100,000 land cover type dataset provided by the Jianfengling Bureau of the Hainan Tropical Rainforest National Park Administration. All environmental variables were extracted to each camera trap site using ArcGIS 10.8 and standardized (mean‐centered, standard deviation scaled). To capture potential non‐linear relationships, NDVI and HII were included in the model as quadratic polynomials, while the other three variables (elevation, aspect, distance to water source) were included as linear terms.

In the R 4.5.0 environment, the JAGS 4.3.1 software was called via the rjags package (Plummer et al. 2016) to construct a Bayesian multi‐species single‐season occupancy model (Clark and Altwegg 2019; Northrup and Gerber 2018; Nguyen et al. 2022). To investigate the influence of seasonal environmental variation on occupancy probabilities and interspecific relationships, we divided the entire camera‐trap dataset into two independent seasons: the dry season (November to April) and the wet season (May to October). Each seasonal dataset comprised its own set of 18 secondary sampling periods of 10 days each and corresponding covariate values. Data were structured into a 3D array [sites × 18 ten‐day occasions × species] (Deng et al. 2021; Fan et al. 2020).

To model a potential asymmetric interaction, we specified that Silver Pheasant occupancy (ψ_SP) was independent of the peacock‐pheasant and only influenced by environmental variables, while Hainan Peacock‐Pheasant occupancy (ψ_HPP) was conditional on the Silver Pheasant's presence state. Detection probability was only influenced by operating duration of cameras, elevation, human influence index, and interspecific interactions (Kéry and Royle 2016; Royle and Dorazio 2008; Richmond et al. 2010).

The Silver Pheasant's occupancy model formula was:

$$z_{\left\{\mathrm{SP},i\right\}}∼\text{Bernoulli}\left(\psi_{\left\{\mathrm{SP},i\right\}}\right)$$

$$\left[\text{logit}\left(\psi_{\mathrm{sp},i}\right)=\alpha_{\mathrm{sp}}+\sum_{k=1}^{4}\beta_{\mathrm{sp},k}\cdot {\text{covar}}_{k,i}+\beta_{\mathrm{sp},\mathrm{hii}-\mathrm{sq}}\cdot {\mathrm{Hii}}_{\text{scaled},i}^{2}+\beta_{\mathrm{sp},\text{ndvi}-\mathrm{sq}}\cdot {\text{NDVI}}_{\text{scaled},i}^{2}\right]$$

The Hainan Peacock‐Pheasant's occupancy model formula was:

$$z_{\left\{\mathrm{HPP},i\right\}}∼\text{Bernoulli}\left(\psi_{\left\{\mathrm{HPP},i\right\}}\cdot z_{\left\{\mathrm{SP},i\right\}}+\psi_{\left\{\mathrm{HPP},i\right\}}^{\left\{\text{absence}\right\}}\cdot \left(1-z_{\left\{\mathrm{SP},i\right\}}\right)\right)$$

$$\left[\text{logit}\left(\psi_{\mathrm{sp},i}\right)=\alpha_{\mathrm{sp}}+\sum_{k=1}^{4}\beta_{\mathrm{sp},k}\cdot {\text{covar}}_{k,i}+\beta_{\mathrm{sp},\mathrm{hii}-\mathrm{sq}}\cdot {\mathrm{Hii}}_{\text{scaled},i}^{2}+\beta_{\mathrm{sp},\text{ndvi}-\mathrm{sq}}\cdot {\text{NDVI}}_{\text{scaled},i}^{2}+\gamma \cdot Z\_\mathrm{bx},i\right]$$

where $z_{s,i}$ is the true occupancy state of species s at site i (1 indicates presence, 0 indicates absence); $\psi_{s,i}$ is the occupancy probability of species s at site i, which is typically modeled via a logit.

where SP represents species, $\alpha_{\mathrm{sp}}$ is the intercept, $\beta_{\mathrm{sp},k}$ are effect parameters for environmental covariates, $\beta_{\mathrm{sp},\mathrm{hii}-\mathrm{sq}}$ and $\beta_{\mathrm{sp},\text{ndvi}-\mathrm{sq}}$ are parameters for the quadratic terms of HII and NDVI respectively, γ·Z_bx,i are interspecific interaction term.

We used vague priors (Normal [0, 4]) for coefficients to minimize subjective bias, provide regularization, and avoid overfitting. Three MCMC chains were run with a 20,000‐iteration burn‐in, followed by 50,000 iterations thinned by 5. Convergence was confirmed (R‐hat < 1.1, ESS > 200) (Gelman et al. 1996; Kéry and Royle 2016). A parameter was considered significant if its 95% Credible Interval (CI) excluded zero (Makowski et al. 2019).

#### 2.3.3 Spatial Interactions

Interspecific spatial association was quantified using the Species Interaction Factor (SIF) derived from the above occupancy model. SIF was calculated as SIF = exp. (γ), with SIF > 1 indicating positive co‐occurrence. We derived SIF for dry and wet seasons separately. Spatial aggregation was assessed using Moran's I statistic on occupancy surfaces generated by inverse distance weighting interpolation. All spatial analyses were conducted in the R environment, primarily using the “sf,” “gstat,” and “spdep” packages (Bivand et al. 2008; Moran 1950; Pebesma 2018; Shukla et al. 2020).

## 3 Results

### 3.1 Temporal Niche

**FIGURE 2:**
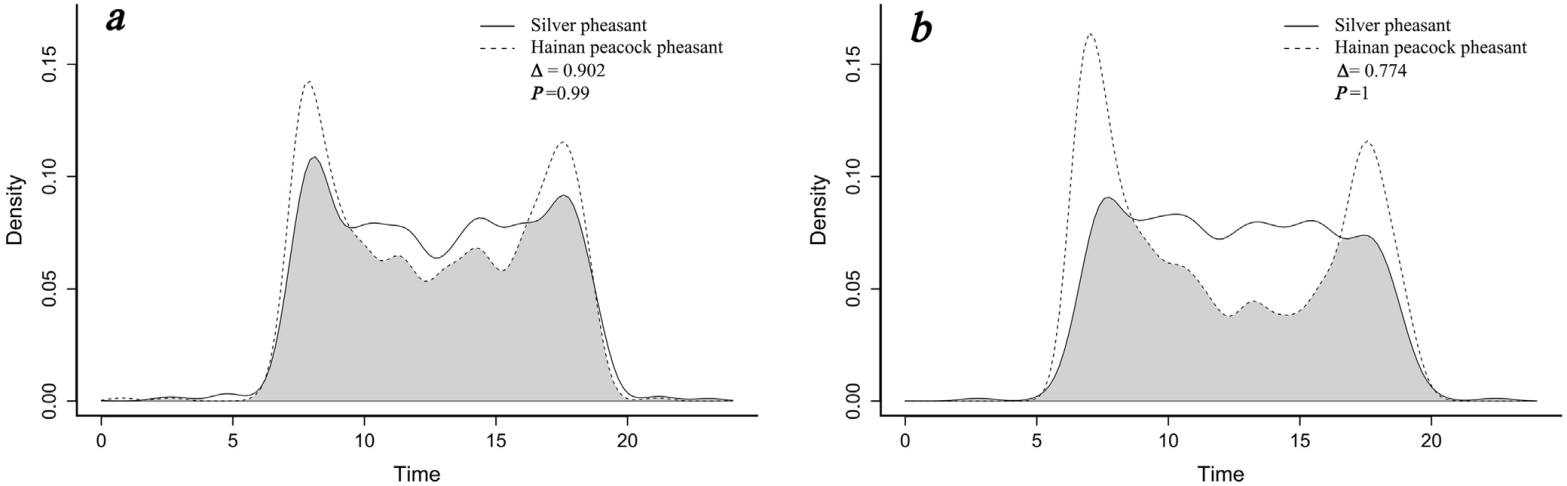
Differences in activity rhythms between dry and wet seasons. Dashed line: Hainan Peacock‐Pheasant; Solid line: Silver Pheasant. Shaded areas indicate the degree of overlap. (a) Dry season, (b) Wet season.

Kernel density analysis revealed that both the Hainan Peacock‐Pheasant and Silver Pheasant are typical diurnal species. The Hainan Peacock‐Pheasant exhibited distinct bimodal activity peaks around dawn (~7:00) and dusk (~18:00) throughout the year (Figure 2). Its activity rhythm differed significantly between dry and wet seasons (overlap Δ = 0.844, *p* < 0.001). The Silver Pheasant showed a pronounced morning peak (~07:00) in the dry season but no distinct crepuscular peaks in the wet season (Figure 2); its seasonal activity patterns were highly similar (Δ = 0.943, *p* > 0.05) (Figure S1). Diel activity overlap between the species was higher in the dry season (Δ = 0.902) than in the wet season (Δ = 0.774). The Peacock‐Pheasant was more active at dawn and dusk, while the Silver Pheasant was more active during midday (Figure 2).

### 3.2 Environmental Drivers of Detection and Occupancy

Detection probability results showed a high degree of consistency between the rainy and dry seasons. The posterior means of the detection intercepts for Silver Pheasant and Hainan Peacock‐pheasant were approximately −1.76 and −3.19, respectively, indicating low baseline detection probabilities for both species, with the detection probability of Hainan Peacock‐pheasant being significantly lower than that of Silver Pheasant. Regarding covariate effects, only the interspecific interaction effect exhibited a significant positive influence in both seasons. In contrast, the effects of camera operating duration, elevation, and the human influence index on detection probability did not reach statistical significance for either species in either season (Table S1).

**FIGURE 3:**
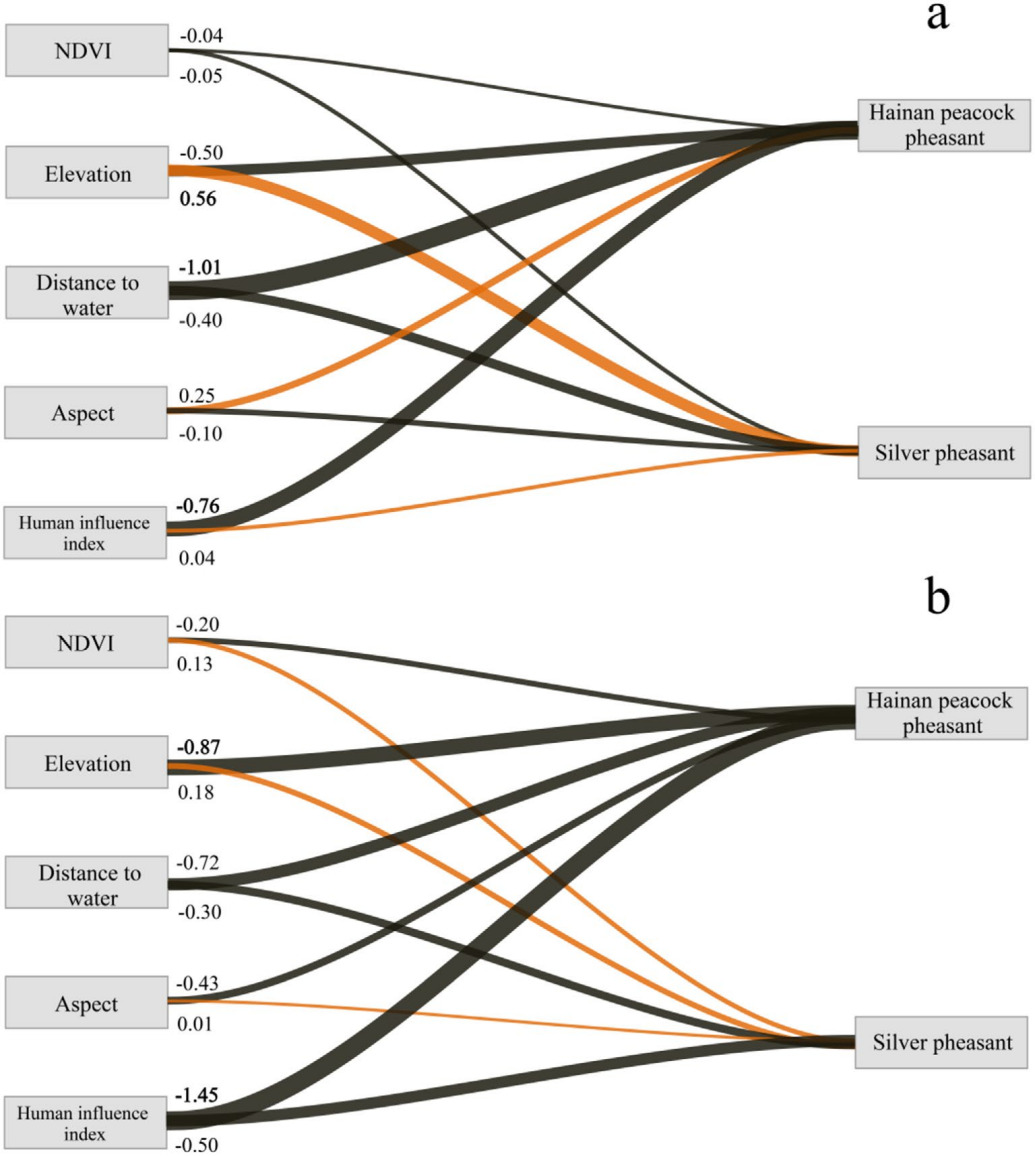
Relationship between environmental factors and occupancy probability of Hainan Peacock‐Pheasant and Silver Pheasant, showing significance and direction of effects. Effect sizes are on the logit scale, representing the effect of a one‐standard‐deviation change in the covariate. Upper values: Hainan Peacock‐Pheasant effects; Lower values: Silver Pheasant effects. Bold values indicate significance. Lines represent median and 95% credible interval. Line width corresponds to effect strength. Positive effects are marked in orange, and negative effects in black‐gold. (a) Dry season, (b) Wet season.

The occupancy model revealed differential effects of environmental variables on the occupancy probability of the two species (Figure 3).

**FIGURE 4:**
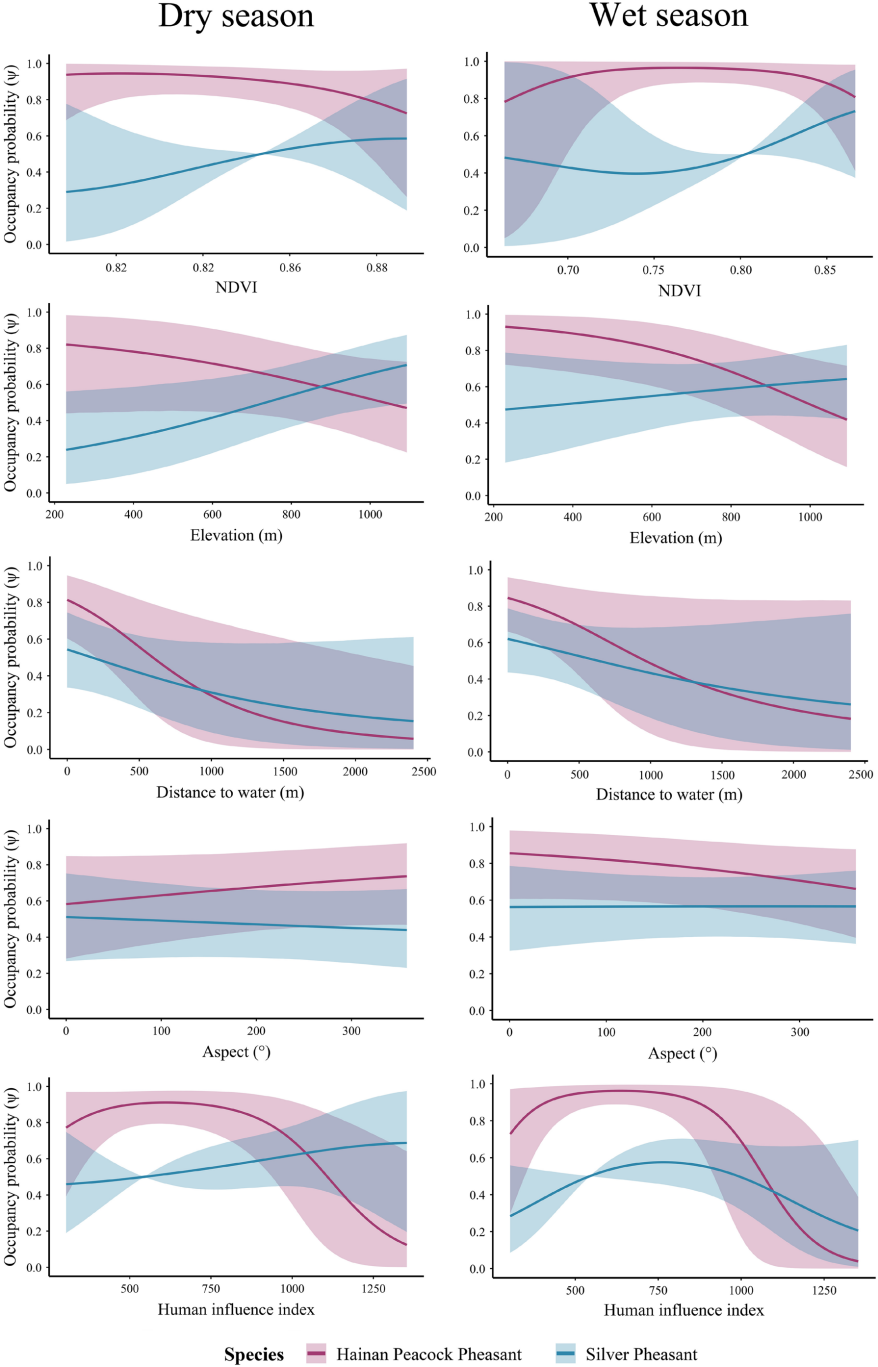
Effect of environmental factors on the occupancy probability of the two pheasant species. Pink solid line: Hainan Peacock‐Pheasant; Blue solid line: Silver Pheasant. Shaded areas represent 95% confidence intervals.

**FIGURE 5:**
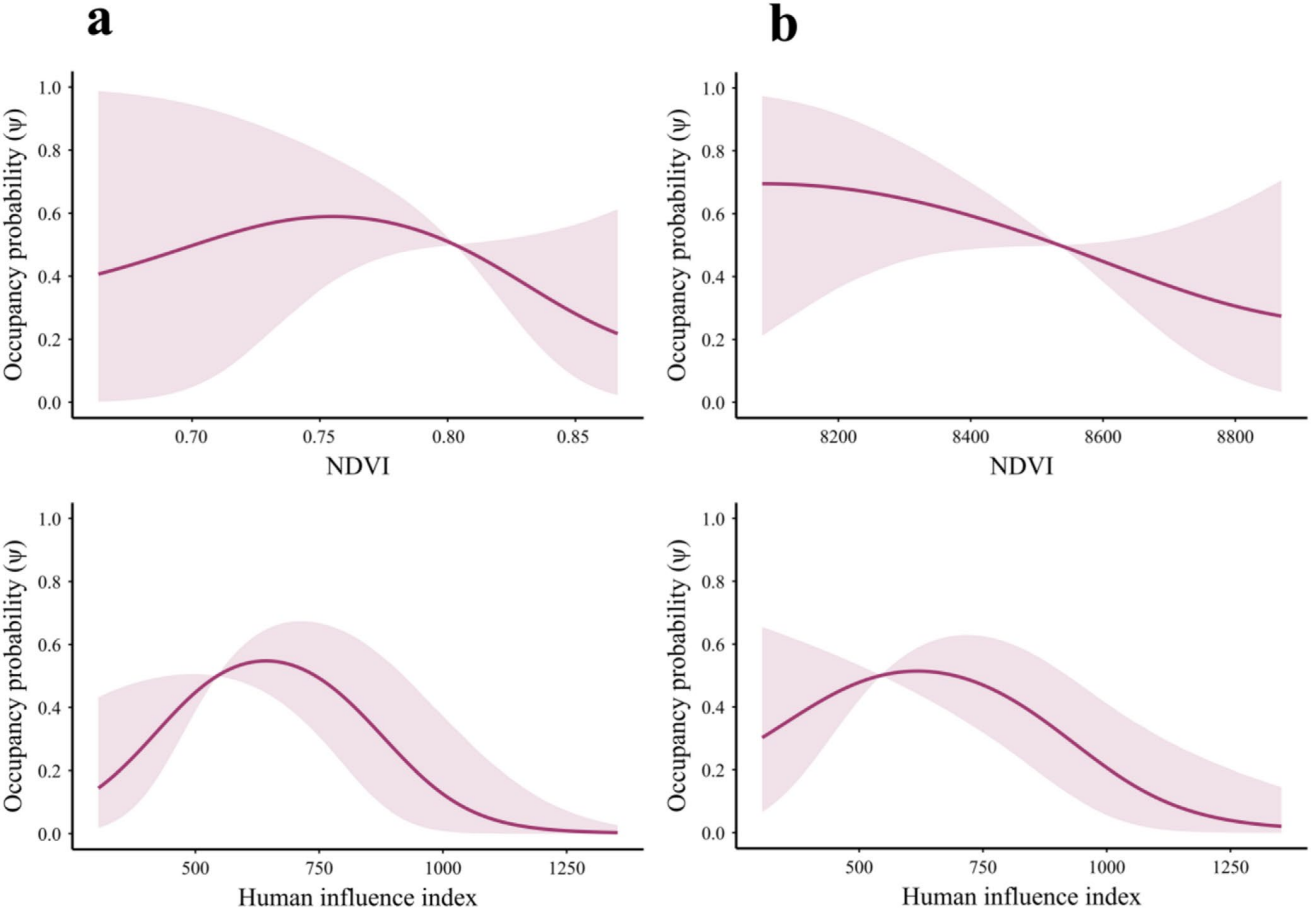
Effect of environmental factors on the occupancy probability of the Hainan Peacock‐Pheasant in the absence of the Silver Pheasant. Pink solid line: Hainan Peacock‐Pheasant. Shaded areas represent 95% confidence intervals. (a) Dry season, (b) Wet season.

For the Hainan Peacock‐Pheasant, occupancy probability was negatively associated with NDVI, especially when co‐occurring with the Silver Pheasant (Figure 4). In the dry season, occupancy declined from 0.95 (95% CI: 0.89–0.98) at NDVI = 0.82 to 0.72 (95% CI: 0.61–0.81) at NDVI = 0.89. This negative relationship was stronger in the wet season; occupancy remained high at 0.96 when NDVI = 0.76 but dropped rapidly to 0.78 when NDVI exceeded 0.85 (Figure 4). In the absence of the Silver Pheasant, its peak occupancy and tolerance for high NDVI were substantially lower (Figure 5). Elevation negatively affected Peacock‐Pheasant occupancy only in the wet season (*β* = −0.87, 95% CI: −1.64 to −0.17). Distance to water negatively influenced the Peacock‐Pheasant's distribution in the dry season only (*β* = −1.01, 95% CI: −1.96 to −0.21). Aspect was not significant for the Peacock‐Pheasant (Figure 3).

The Hainan Peacock‐Pheasant's occupancy probability showed a significant negative relationship with HII. When co‐occurring with the Silver Pheasant, its occupancy peaked at HII ≈611 (0.91) in the dry season, plummeting to 0.12 at HII ≈1353. In the wet season, it peaked at HII ≈632.5 (0.96), dropping sharply to 0.04 at HII ≈1353 (Figure 4). When the Silver Pheasant was absent, peak occupancy was lower and approached zero in high‐disturbance areas (Figure 5).

For the Silver Pheasant, the relationship with NDVI shifted from weakly unimodal in the dry season to positive in the wet season (Figure 4). Silver Pheasant occupancy was positively associated with elevation in the dry season (*β* = 0.56, 95% CI: 0.06–1.10). Distance to water had no significant effect on the Silver Pheasant. Aspect was not significant for the Silver Pheasant (Figure 3). The Silver Pheasant exhibited a different response to disturbance: occupancy increased with higher HII in the dry season, highest (0.69) at HII ≈1353; in the wet season, occupancy was negatively correlated with HII, peaking (0.57) at HII ≈770 and dropping to 0.21 at HII ≈1353 (Figure 4).

### 3.3 Spatial Association

The HBOM indicated a significant positive spatial association between the Hainan Peacock‐Pheasant and Silver Pheasant, which was stronger in the wet season (SIF = 3.01, 95% CI: 1.94–4.93) than in the dry season (SIF = 2.48, 95% CI: 1.34–3.64). Spatial analysis (Moran's I) confirmed that both species' distributions were highly aggregated (proportion of aggregated sites: 97.6% dry season, 96.8% wet season). The proportion of sites where both species co‐occurred increased from 9.6% in the dry season to 12.8% in the wet season.

## 4 Discussion

Our integrated analysis reveals that the coexistence of the endangered endemic Hainan Peacock‐Pheasant and the more common Hainan Silver Pheasant is facilitated by multidimensional niche partitioning but is critically mediated by human disturbance within the typical tropical montane rainforest habitat of Jianfengling, Hainan. This dynamic coexistence shifts from a stable, differentiated state in intact forest to a fragile, conditional association under anthropogenic pressure, where the Hainan Peacock‐Pheasant's occupancy collapses at high disturbance levels while the Silver Pheasant's occupancy peaks at intermediate disturbance; their positive spatial association occurs only in low‐to‐moderate disturbance areas, and their temporal niche separation is stronger in the wet season than in the dry season.

### 4.1 Multidimensional Niche Partitioning Facilitates Coexistence

Firstly, along the temporal dimension, the separation of activity peaks is a key behavioral strategy to reduce direct competition (Chesson 2000). The Hainan Peacock‐Pheasant's typical bimodal dawn/dusk activity pattern contrasts sharply with the Silver Pheasant's relatively flat, plateau‐like diurnal activity, especially showing clear avoidance during critical dawn and dusk periods. Although the two species exhibited similar activity patterns within each season. This differentiation is not static but exhibits dynamic seasonal adaptation: activity overlap was substantially higher in the dry season (Δ = 0.902) than in the wet season (Δ = 0.774). This strongly suggests that during the resource‐scarce dry season, both species may be forced to expand their activity windows to meet energy demands, leading to temporary constriction of temporal niches. When resource abundance increases in the wet season, competitive pressure eases, allowing them to fully execute their inherent, more distinctly differentiated activity strategies (Gause 1934; Kronfeld‐Schor and Dayan 2003). The intensified crepuscular activity of the Peacock‐Pheasant in the wet season may be an adaptation to exploit optimal microclimates and peak invertebrate activity (Gao 1996; Liang and Zhang 2011).

Secondly, along the spatial dimension, differences in vertical distribution and microhabitat selection form the physical basis for coexistence. Occupancy model results show that the two species exhibited near “mirror‐image” preferences for elevation and NDVI. The Silver Pheasant favored higher elevations with high NDVI (dense canopy), congruent with its omnivorous, shrub‐layer foraging (Taylor et al. 2021). Conversely, the Hainan Peacock‐Pheasant significantly prefers mid‐to‐low elevation habitats with medium‐to‐low NDVI (relatively open understory). This reliance on open understory likely stems from its specialized ground‐foraging strategy, requiring some understory light to locate invertebrates and fallen fruits, as overly dense vegetation impedes movement and prey detection (Liu et al. 2013). This complementary use of vertical and vegetation‐structure space minimizes direct competition, exemplifying classic niche differentiation (MacArthur 1958).

Finally, differences in demand for key resources (e.g., water) further refine niche boundaries. The Hainan Peacock‐Pheasant showed significant dependence on water sources during the dry season, consistent with the behavior of many tropical terrestrial birds in dry periods, reflecting physiological water needs or reliance on food aggregated in microhabitats near water (Brandt and Cresswell 2008; Smith et al. 2010). In contrast, the Silver Pheasant showed no significant response to distance from water, indicating a broader ecological amplitude and stronger water metabolism adaptability. This difference in resource specialization is one intrinsic reason for their differing vulnerability to environmental change.

### 4.2 Conditional Positive Association Modulated by Human Disturbance

A key finding was the significant positive spatial association (SIF > 1), which intensified in the wet season. This contrasts with expectations from the classic competitive exclusion principle (Gause 1934), yet opens a new perspective for understanding species interactions in complex tropical communities.

This positive association may be explained by several non‐exclusive mechanisms. First, the two species may share broad habitat requirements. Although they exhibit fine‐scale niche differentiation, both species depend on intact montane rainforest at the landscape scale. High‐quality forest patches in Jianfengling are likely to provide the core resources needed by both species, resulting in a greater probability of co‐occurrence than would be expected by chance alone. Second, facilitative interactions or indirect positive effects may be at play. The Silver Pheasant's larger body size presumably enables substantial physical disturbance of the leaf litter layer during foraging, which may inadvertently expose soil invertebrates or scatter fruits, thereby enhancing foraging conditions for the Hainan Peacock‐Pheasant. While this process is conceptually consistent with “ecosystem engineering” (Michel et al. 2020), it remains to be empirically tested. Third, both species exhibit convergent behavioral responses to common threats. They share predators and show similar avoidance behaviors in response to human activity and disturbance, leading to spatial aggregation within shared refuge areas (Lima and Bednekoff 1999; Li 2005; Zeng 2025).

Critically, human disturbance acts as a powerful filter on this interaction. The Peacock‐Pheasant exhibited extreme sensitivity, with occupancy collapsing in high‐disturbance areas. In contrast, the Silver Pheasant showed a response consistent with the intermediate disturbance hypothesis (Connell 1978). This disparity creates a conservation paradox: in pristine forests, niche partitioning supports stable coexistence, but under anthropogenic pressure, the relationship becomes asymmetrically dependent, with the sensitive Peacock‐Pheasant disproportionately affected. The positive association persisted only in low‐to‐moderate disturbance areas and broke down completely under high disturbance. Thus, the Silver Pheasant's adaptability does not confer an “umbrella” effect for its more sensitive counterpart under intense pressure; instead, the coexistence mechanism itself is destabilized.

### 4.3 Conclusions and Conservation Implications

The coexistence of the Hainan Peacock‐Pheasant and Silver Pheasant is a delicate balance maintained by fine‐scale niche partitioning, a balance highly vulnerable to human encroachment. Effective conservation must be spatially explicit and informed by these mechanistic insights. We recommend: (1) prioritizing core habitat protection for the Hainan Peacock‐Pheasant, focusing on primary forests at 500–800 m with open understory and proximity to water; (2) implementing a tiered buffer system around core zones, where inner buffers maintain minimal disturbance and outer buffers allow strictly regulated, low‐impact activities; (3) maintaining landscape connectivity and elevation gradients to ensure genetic exchange and provide climate refugia; and (4) integrating mechanistic metrics (e.g., activity overlap, SIF) into long‐term monitoring to track the health of this interspecific dynamic. Our study underscores that protecting biodiversity requires understanding and preserving the intricate interactions that sustain it.

## Author Contributions

**Xiangxiang Lu:** conceptualization (equal), investigation (equal), methodology (equal), software (lead), validation (equal), visualization (lead), writing – original draft (lead), writing – review and editing (equal). **Wenbo Yan:** conceptualization (equal), data curation (equal), formal analysis (equal), funding acquisition (equal), investigation (equal), methodology (equal), project administration (equal), resources (equal), supervision (lead), validation (equal), writing – original draft (supporting), writing – review and editing (equal). **Zhigao Zeng:** project administration (equal), resources (equal), supervision (equal), writing – review and editing (equal). **Shaoliang Xue:** investigation (equal), resources (equal). **Qi Wang:** data curation (equal), formal analysis (equal), supervision (equal).

## Funding

This research was funded by the Hainan Institute of National Park, grant numbers: KY‐24ZK01 and KY‐21ZK01, and the Jianfengling Branch Bureau of Hainan Tropical Rainforest National Park Management Office, grant number: Donghe (Ke) 2020 – 0312.

## Ethics Statement

This work did not require ethical approval from a human subject or animal welfare committee. We received the required research permits to work in protected areas from Jianfengling Branch Bureau of Hainan Tropical Rainforest National Park Management Office.

## Conflicts of Interest

The authors declare no conflicts of interest.

## Supporting information


**Figure S1:** Differences in activity rhythms between Hainan peacock‐pheasant and white pheasant during dry and rainy seasons. Dashed lines represent the rainy season, while solid lines represent the dry season. The shaded areas indicate the degree of overlap (Δ). *p*‐value indicates statistical significance.
**Table S1:** Posterior means and 95% credible intervals of occupancy probability (ψ) and detection probability (*p*) for the two pheasant species in dry and wet seasons.

## Data Availability

https://doi.org/10.5281/zenodo.20379271.
