# Supplementary material for: Niche Partitioning and Facilitative Coexistence of Two Sympatric Pheasants Across a Gradient of Human Disturbance
Source: Ecol Evol. 2026 Jul 2;16(7):e73929. doi: 10.1002/ece3.73929 (PMC13327799; doi:10.1002/ece3.73929)
Supplement: Supplementary file 1 — Figure S1: Differences in activity rhythms between Hainan peacock‐pheasant and white pheasant during dry and rainy seasons. Dashed lines represent the rainy season, while solid lines represent the dry season. The shaded areas indicate the degree of overlap (Δ). p‐value indicates statistical significance. Table S1: Posterior means and 95% credible intervals of occupancy probability (ψ) and detection probability (p) for the two pheasant species in dry and wet seasons. [file ECE3-16-e73929-s001.doc]

**Table S1** Posterior means and 95% credible intervals of occupancy probability (ψ) and detection probability (p) for the two pheasant species in dry and wet seasons.

| **Covariates** | **Wet Season** | | | | **Dry Season** | | | | | |  | | |
| --- | --- | --- | --- | --- | --- | --- | --- | --- | --- | --- | --- | --- | --- |
|  | **Hainan Peacock Pheasant** | | **Silver Pheasant** | | **Hainan Peacock Pheasant** | | | **Silver Pheasant** | | |  | | |
| **Occupancy** | | | | | | | | | | |  | | |
| Intercept | **-2.021(-3.394,-0.836)** | | 0.271(-0.403,0.979) | | **-1.607(-2.81,-0.522)** | | | | -0.129(-0.918,0.668) | | |  | |
| Elevation | **-0.870 (-1.64,-0.173)** | | 0.181 (-0.302,0.675) | | -0.501 (-1.164, 0.126) | | | 0.561 (-0.055, 1.089) | | |  | | |
| Aspect | -0.429 (-1.060,0.161) | | 0.005 (-0.425,0.428) | | 0.252 (-0.221,0.745) | | | -0.101 (-0.517,0.311) | | |  | | |
| Distance from water | -0.722 (-1.636, 0.003) | | -0.301 (-0.821, 0.148） | | **-1.007 (-1.96,-0.213)** | | | -0.401 (-0.977,0.085） | | |  | | |
| NDVI | -0.215(-0.666-0.207) | | 0.132(-0.266,0.590) | | -0.039(-0.35,0.269) | | | -0.046(-0.374,0.261) | | |  | | |
| [Human Influence Index](https://www.researchgate.net/figure/Human-Influence-Index-HII-Version-2-accessed-via-the-Socioeconomic-Data-and_fig1_340331517) | **-1.448 (-2.561,-0.499)** | | -0.449 (-1.035,0.096） | | **-0.762（-1.552,-0.043）** | | | | 0.039(-0.501,0.575) | | |  | |
| **Detection** | | | | | | | | | | |  | | |
| Intercept | **-3.19（-4.802-1.936）** | | | **-1.762(-1.967,-1.569)** | | **-3.185（-4.764,-1.936）** | | | | **-1.762(-1.963,-1.567)** | | |  |
| Elevation | -0.045(-0.307,0.217) | | 0.04(-0.155,0.236) | | -0.047(-0.304,0.215) | | | 0.039(-0.153,0.236) | | |  | | |
| Operating duration of cameras | -0.133(-0.368,0.097) | | -0.161(-0.352,0.029) | | -0.133(-0.374,0.094) | | | -0.161(-0.354,0.031) | | |  | | |
| Human Influence Index | -0.246(-0.763,0.202) | | -0.175(-0.467,0.086) | | -0.2245(-0.762,0.206) | | | -0.173(-0.466,0.085) | | |  | | |
| Interspecific relationships | | **1.81(0.545,3.384)** | | | | | **1.805(0.543,3.369)** | | | | | | |

The 95% credible intervals appear in parentheses. Variables not including zero in the credible interval appear in boldface.


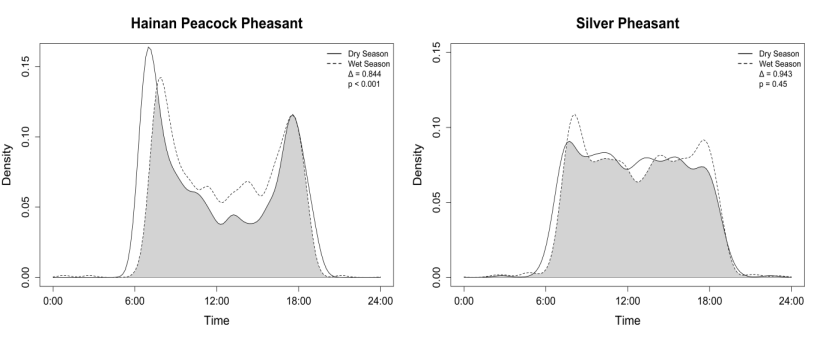


**Figure S1**: Differences in activity rhythms between Hainan peacock-pheasant and white pheasant during dry and rainy seasons. Dashed lines represent the rainy season, while solid lines represent the dry season. The shaded areas indicate the degree of overlap (Δ). P-value indicates statistical significance.
